# Ingested *Salmonella enterica, Cronobacter sakazakii, Escherichia coli* O157:H7*,* and *Listeria monocytogenes*: transmission dynamics from adult house flies to their eggs and first filial (F_1_) generation adults

**DOI:** 10.1186/s12866-015-0478-5

**Published:** 2015-07-31

**Authors:** Monica Pava-Ripoll, Rachel E. Goeriz Pearson, Amy K. Miller, Ben D. Tall, Christine E. Keys, George C. Ziobro

**Affiliations:** U.S. Food and Drug Administration, Center for Food Safety and Applied Nutrition, Office of Food Safety, 5100 Paint Branch Pkwy, College Park, MD 20740 USA; U.S. Food and Drug Administration, Center for Food Safety and Applied Nutrition, Office of Applied Research and Safety Assessment, 8301 Muirkirk Rd, Laurel, MD 20708 USA; U.S. Food and Drug Administration, Center for Food Safety and Applied Nutrition, Office of Regulatory Science, 5100 Paint Branch Pkwy, College Park, MD 20740 USA

## Abstract

**Background:**

The mechanical transmission of pathogenic bacteria by synanthropic filth flies is widely recognized. While many studies report the fate and the temporospatial distribution of ingested foodborne bacteria by filth flies, there is little evidence about the transmission dynamics of ingested foodborne bacteria by adult house flies (*Musca domestica*) to their progeny. In this study, we fed parental house fly adults with food contaminated with low, medium, and high concentrations of *Salmonella enterica*, *Cronobacter sakazakii*, *Escherichia coli* O157:H7*,* and *Listeria monocytogenes* and evaluated the probability of transmission of these pathogens to house fly eggs and the surface and the alimentary canal of their first filial (F_1_) generation adults.

**Results:**

All foodborne pathogens were present in samples containing pooled house fly eggs. The probability of transmission was higher after parental house flies ingested food containing medium bacterial loads. *Cronobacter sakazakii* was 16, 6, and 3 times more likely to be transmitted to house fly eggs than *S. enterica*, *E. coli* O157:H7, and *L. monocytogenes*, respectively. Only *S. enterica* and *C. sakazakii* were transmitted to F_1_ generation adults and their presence was 2.4 times more likely on their body surfaces than in their alimentary canals. The highest probabilities of finding *S. enterica* (60 %) and *C. sakazakii* (28 %) on newly emerged F_1_ adults were observed after parental house flies ingested food containing medium and high levels of these pathogens, respectively.

**Conclusion:**

Our study demonstrates that adult house flies that fed from food contaminated with various levels of foodborne bacteria were able to transmit those pathogens to their eggs and some were further transmitted to newly emerged F_1_ generation adults, enhancing the vector potential of these insects. Understanding the type of associations that synanthropic filth flies establish with foodborne pathogens will help to elucidate transmission mechanisms and possible ways to mitigate the spread of foodborne pathogens.

**Electronic supplementary material:**

The online version of this article (doi:10.1186/s12866-015-0478-5) contains supplementary material, which is available to authorized users.

## Background

The biology and ecology of synanthropic insects like flies make them efficient carriers of disease-causing microorganisms. Their breeding habits, mode of feeding and indiscriminate traveling between decomposed waste and human settings highly contribute to the dissemination of pathogens in the environment. Approximately 350 fly species in 29 families are potentially associated with the transmission of diseases of public health importance [1]. However, fewer numbers of fly species have been associated with the transmission of foodborne pathogens [1, 2]. Although there are scarce reports of filth flies being the causative agent of foodborne outbreaks, several studies have demonstrated a steady decrease in the incidence of foodborne diarrhea after suppressing fly populations [3–5], indirectly implicating filth flies as the source of the foodborne pathogen.

The presence of flies in food and food facilities has always been a concern of the U.S. Food and Drug Administration (FDA). The FDA’s regulatory action criteria for filth includes a five-attribute profile that needs to be fulfilled before including a particular fly species as reasonably likely to act as a contributing factor of the spread of foodborne pathogens. These five attributes are synanthropy, endophily, communicative behavior, attraction to filth and human food, and the isolation of pathogens from wild populations [1, 2]. Other fly species fulfilling at least four of those attributes are considered opportunistic pests and their presence in food and/or food-related environments is an indication of insanitation [2].

Foodborne pathogens transmitted by synanthropic filth flies are found not only externally on the fly surface (which includes body, head, legs, and wings), but also internally, mainly in the alimentary canal (which runs the length of the body, from pharynx to anus) [6]. In fact, we previously reported that foodborne pathogens were up to three times more likely to be found in the alimentary canal than on the body surface of wild flies caught in and around urban restaurant dumpsters [7]. Consequently, flies can contaminate food or food-contact surfaces mechanically or through regurgitation or defecation. The potential spread of foodborne pathogens increases when there is a focus of infection for a particular bacterium [8]. Our previous study showed a statistically significant association between the presence of *Salmonella enterica*, *Listeria monocytogenes* and *Cronobacter* spp. (former *Enterobacter sakazakii*) on the surface and in the guts of wild flies and the sites where those flies were collected [7]; thus, emphasizing that bacteria inhabiting the alimentary canal of flies are acquired from the surrounding environment. Filth flies also travel quickly and may move several miles [9]; therefore, they can rapidly intensify the risk of foodborne diseases by transporting pathogens from places where the pathogens pose no hazard to places where they do, such as food preparation areas [1].

The transmission process of a particular pathogen in populations of synanthropic filth flies determines the spread and persistence of that pathogen. Thus, information about the transmission dynamics of a particular pathogen within a fly population is essential to appropriately avoid the spread of foodborne diseases. It is important to note that understanding the epidemiology of an illness caused by a pathogen transmitted by flies, requires a deeper knowledge of the ecology, physiology, immunology, and genetics of the pathogen as well as the morphology, physiology, and behavior of the fly. Nevertheless, it is even more important to understand how pathogen and fly interact in a particular environment [10, 11].

Filth flies can be transient or definitive hosts of pathogens and, like vertebrates, they may be immune or susceptible to infection. Although flies can internally harbor foodborne bacteria, it is not well known if these pathogens are beneficial or harmful to them. However, flies have shown remarkable resilience to these pathogens. For instance, several species of *Cronobacter* have been isolated from the alimentary canal of several flies collected in the wild [7, 12–16] and have also shown to support the development of stable fly larvae in the absence of other microbes, by colonizing the alimentary canal of newly emerged flies [16]. Flies have also shown efficient and rapid responses to ingested *Escherichia coli* O157:H7 since excretion of this pathogen was observed 6 to 24 h after being ingested [17, 18].

There are plenty of studies reporting the mechanical transmission of foodborne pathogens by filth flies (some examples include [7, 19–22]) and there are other studies reporting the fate and the temporospatial distribution of ingested foodborne pathogens by flies [17, 18, 23–26]. However, there is little scientific information about the transmission dynamics of foodborne bacteria to the fly’s progeny after parental flies have ingested those pathogens. The objective of this study was to estimate the probability of transmission of four foodborne bacteria (*S. enterica*, *C. sakazakii*, *E. coli* O157:H7*,* and *L. monocytogenes* ) to the progeny of the common house fly, *Musca domestica* (Linneaus) (Diptera: Muscidae), after parental house flies were fed with food contaminated with low, medium, and high levels of each bacterium. The presence of each pathogen was evaluated on pooled house fly eggs laid by parental females and on the surface and in the alimentary canal of newly emerged first filial (F_1_) generation adults.

## Results and discussion

All parental house flies used in our experiments were observed feeding from contaminated food and the presence of each pathogen was confirmed from all alimentary canals dissected from randomly selected parental females. Although the focus of this study was not to evaluate the dynamics of the parental population of adult house flies, anecdotal evidence suggests that the feeding and mating behaviors were not influenced by the ingestion of bacteria, and although not measured, we did not observe apparent increases in the mortality of parental flies or reductions in their oviposition rate, when compared to control groups. We observed clusters of house fly eggs on the oviposition substrate approximately 10-16 h after they were placed on the mesh of all jars.

The combined molecular and culture approach that we used to detect and isolate the foodborne pathogens from samples of pooled house fly eggs, and the surfaces and alimentary canals of single adult house flies was straightforward when evaluating for the presence of *S. enterica, E. coli* O157:H7, and *L. monocytogenes.* We easily obtained pure colonies of these three pathogens from the enrichment media of all PCR-positive samples. Likewise, we easily obtained pure *C. sakazakii* colonies from the enrichment media of all PCR-positives from pooled house fly eggs and alimentary canals of parental flies. However, the isolation of this pathogen from PCR-positive samples from F_1_ adults was more challenging and required several subculturing steps on selective media. Consequently, we could only obtain pure colonies of *C. sakazakii* from eight out of 15 PCR-positive samples from F_1_ adults.

We have previously reported that *C. sakazakii* colonies could not be recovered from some PCR-positive samples while using this combined approach, likely due to the PCR being positive when other closely related bacterial genera*,* such as *Citrobacter freundii*, are present in the samples [7, 27]. Additionally, besides *C. sakazakii* a number of other *Enterobacteriaceae* are α-glucosidase positive, therefore the co-isolation of those organisms from samples with highly complex microbiota (such as the fly’s alimentary canal) could lower the efficiency of recovery of *C. sakazakii* from the chromogenic media used [28]. As a result, only those samples from which pure *C. sakazakii* colonies were isolated, were considered positive for the presence of the pathogen and included for statistical analysis. No pathogens were observed on chromogenic media from any of the PCR-negative samples that were randomly selected.

Pure colonies of *S. enterica*, *L. monocytogenes*, and *E. coli* O157:H7 isolated from PCR-positive samples were confirmed to be identical to the strains ingested by parental house flies by showing indistinguishable PFGE profiles (see Additional file 1). Likewise, matching nucleotide sequences were obtained from pure colonies of *C. sakazakii* when performing nucleotide comparison of the amplified fragment (463 bp) of the *cgcA C. sakazakii* gene.

### Probability of bacterial transmission to house fly eggs

Our study reports the probability of the presence of the target pathogen in a sample containing pooled house fly eggs laid by several females fed from contaminated food. This study does not attempt to report the transmission rate of individual eggs laid by one or several female flies. The stepwise selection model of the logistic regression analysis indicated that the predicted probability of the presence of bacteria in samples of pooled house fly eggs was associated with the type of foodborne pathogen and the level of bacterial contamination of the food given to parental house flies. However, there was not a significant interaction between these two variables; thus, the interaction was removed from the full model described in Eq. 1. The model fit statistics and the AUC value of 0.89 (excellent discrimination) shows that our data fit the model relatively well. Results from the analysis of the maximum likelihood estimates of the parameters included in the logistic regression model and the model fit statistics for house fly eggs are included in Additional file 2(A).

For all bacteria evaluated, there was a higher chance of the presence of the pathogen in samples with house fly eggs after parental flies received food containing medium levels of bacteria (Table 1A). In fact, when parental house flies received food containing medium bacterial loads, the pathogens were two and six times more likely to be present in the samples than when parental flies fed from food contaminated with high and low bacterial levels, respectively. Therefore, there was not a positive correlation between the levels of contaminated food given to parental flies and the presence of the pathogens in samples with pooled house fly eggs.

**Table 1:** Odds ratios estimates of the presence of foodborne pathogens

	Foodborne pathogen	Bacterial levels in food	Fly’s body part	Odds ratio (95 % CL)
A) House fly eggs				
		Medium vs. high		1.9 (0.5, 6.8)
		Medium vs. low		6.0 (1.7, 20.4)
		High vs. low		3.2 (1.1, 9.6)
	*C. sakazakii* vs. *S. enterica*			15.5 (2.9, 82.6)
	*C. sakazakii* vs. *E. coli* O157:H7			5.7 (1.0, 31.3)
	*C. sakazakii* vs. *L. monocytogenes*			3.0 (0.5, 16.4)
	*L. monocytogenes* vs. *S. enterica*			5.2 (1.5, 18.7)
	*L. monocytogenes* vs. *E. coli* O157:H7			1.9 (0.5, 7.2)
	*E. coli* O157:H7 vs. *S. enterica*			2.7 (0.8, 8.6)
B) F_1_ female adults				
	*S. enterica*	Medium vs. high		2.4 (1.7, 3.4)
			Surface vs. alimentary canal	2.4 (1.7, 3.4)
	*C. sakazakii*	High vs. medium		2.2 (1.3, 3.5)
			Surface vs. alimentary canal	2.4 (1.5, 3.8)

(A) house fly eggs and (B) first filial (F_1_) generation adults

The transmission potential of ingested bacteria to the house fly progeny is a very complex process. Flies harbor many microorganisms (including human pathogens) in their alimentary canals and they require the ingestion of live bacteria for their development. However, feeding from contaminated food does not imply that flies will become infected themselves or that ingested pathogens will survive, proliferate, and/or invade the reproductive system to be transovarially transmitted to house fly eggs and to subsequent life stages or generations. House flies can fight ingested opportunistic invaders by using physical barriers (*i.e*. the type II peritrophic matrix of the midgut epithelium), physiological defenses (*i.e.* digestive processes: pH, and digestive enzymes such as lysozyme), and innate immune response (*i.e.* the secretion of antimicrobial peptides, AMPs, by the fat body) [23, 29]. House flies also carry symbiotic bacteria from one source to another and from one generation to the next [30]. Other studies have suggested that the presence of inherited symbiotic bacteria in insects increases the insect’s resistance to pathogens; thus, inherited symbionts may have important effects on the ecology and evolutionary dynamics of host-pathogen interactions [31–33]. For instance, symbiotic *Klebsiella oxytoca* has been associated with house fly eggs. This bacterium is deposited on the surface of the eggs, inducing female oviposition. However, when *K. oxytoca* is above the threshold abundance levels, it causes oviposition inhibition [34]. Nonetheless, the threshold levels of many other ingested bacteria that will trigger a particular defense mechanism (s) or particular behaviors in the house fly are not yet well known. Nayduch and Joyner [35] detected lysozyme protein in adult house flies that ingested 1.2×10^5^ CFU/μl of *Staphylococcus aureus*, and in their life history stages (eggs, larval instars, and F_1_ adults), providing evidence that the digestive and defensive dual role of lysozymes was activated by the ingestion of high levels of these bacteria. These facts could help to explain the lower rates of contamination found in samples containing pooled house fly eggs laid by females that ingested high levels of contaminated food. However, more research is needed to determine the role of specific foodborne bacteria in house flies and the threshold levels that will trigger defense mechanisms or behaviors in these insects.

**Fig. 1:**
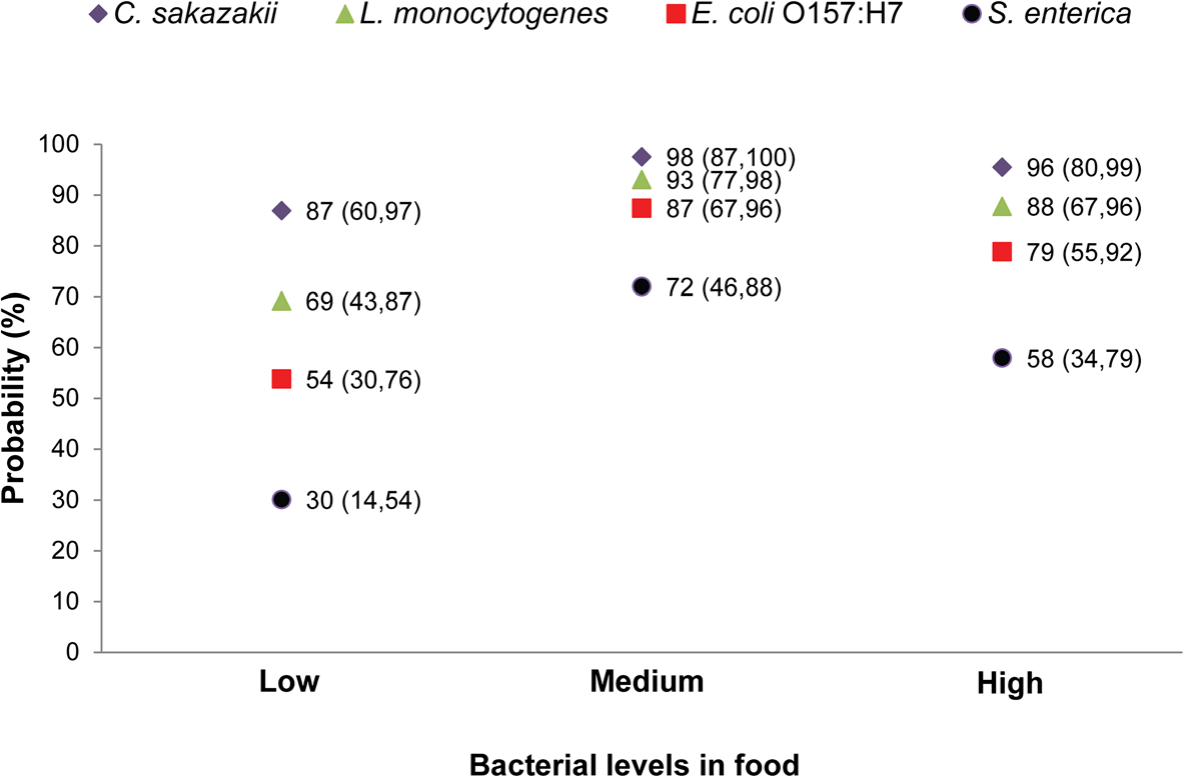
Probability of bacterial transmission to house fly eggs. Numbers in parenthesis represent lower and upper 95 % confidence limits (CL)

The highest rates of contamination of house fly eggs were observed when parental flies fed from food contaminated with *C. sakazakii* (Fig. 1). Percentages of contamination of 87, 98, and 96 % were observed after parental house flies fed from food containing low, medium, and high levels of *C. sakazakii*, respectively. This was followed by the ingestion of food contaminated with *L. monocytogenes* and *E. coli* O157:H7. The contamination rate of house fly eggs with *S. enterica* was lower than other pathogens: 30, 72, and 58 %, after parental house flies received food containing low, medium, and high levels of this pathogen, respectively (Fig. 1). Regardless of the level of contamination of the food given to parental house flies, *C. sakazakii* was 16, 6, and 3 times more likely to contaminate house fly eggs than *S. enterica*, *E. coli* O157:H7, and *L. monocytogenes*, respectively. Similarly, *L. monocytogenes* was 5 and 2 times more probable to contaminate house fly eggs than *S. enterica* and *E. coli* O157:H7, respectively (Table 1A).

Although the groups of collected house fly eggs were surface-disinfected and we obtained no bacterial growth from aliquots of water from the last rinse of the surface-disinfection process, this only demonstrates that no more bacteria could be dislodged from the surface of the eggs (also known as chorion). To verify that bacterial cells were not adsorbed onto the surface of house fly eggs, we randomly selected several surface-disinfected eggs and used them either to take scanning electron microscopy (SEM) images or to plate them on the surface of chromogenic media specific for each pathogen. Even though SEM images of individual eggs did not reveal the attachment of bacterial cells to the chorion (see Additional file 3), we observed the presence of typical bacterial colonies surrounding some of the surface-disinfected house fly eggs that were individually plated. Thus, indicating that some bacterial cells remained attached to the chorion of surface-disinfected eggs.

Ingested bacteria could be adsorbed onto the surface of house fly eggs during or after oviposition because in female house flies the vaginal opening is in close proximity to the anal opening [36], which may facilitate contamination of the egg’s surface with waste products of the fly’s digestive tract. Bacterial cells could remain attached to the chorion due to the adhesive fluid that covers the eggs when they are laid. This fluid is secreted by the accessory glands of the female reproductive system and causes the eggs to adhere to each other and to the material where they were laid [37]. Additionally, the chorionic sculpture of house fly eggs has minute hexagonal markings, distinct curved rib-like thickenings (the hatching line), and some elevations and depressions [36, 37] that could hinder the dislodgement of bacterial cells during the surface-disinfection process.

We did not perform histological studies or transmission electron microscopy (TEM) to demonstrate the presence and/or possible development of the target bacteria in the internal tissues of the eggs. Thus, in this study we cannot confirm that the presence of pathogens in samples containing pooled house fly eggs was due to the transfer of bacteria at early stages of oogenesis and embryogenesis, as required during true transovarial transmission. Instead, the presence of pathogens in samples with pooled house fly eggs was probably due to the adsorption of bacterial cells onto the surface of the eggs during or after oviposition. Bacteria adsorbed on the surface of the eggs can proliferate in the larval rearing substrate and re-contaminate the hatching larvae, creating new focus of infection from where the newly hatched larvae can re-acquire the pathogen. In fact, random samples from larval rearing substrates taken the same day that pupae were removed from the rearing chambers evidenced the presence of the target pathogens (data not shown). Bacteria associated with house fly eggs have been found to supplement the rearing substrate of the developing larvae [38]. However, in this study we did not evaluate the presence of pathogens in any of the F_1_ larval stages. Future studies in our lab will assess the temporospatial fate of green fluorescent protein (GFP)-expressing *S. enterica* and/or *C. sakazakii* from individual eggs laid by female house flies fed with contaminated food. We will also evaluate the presence of the pathogen on the surface and internal tissues of the developing stages of the house fly (three larval instars, puparia, and newly-emerged adults) to have a better understanding of the trans-stadial transmission of those pathogens during metamorphosis.

### Probability of bacterial transmission to house fly F_1_ adults

House fly F_1_ adults were observed in all treatments, indicating the successful completion of the house fly’s life cycle. No pathogens were detected from the surface or the alimentary canal of any of the adult specimens that were sampled from the control groups. Even though *L. monocytogenes* and *E. coli* O157:H7 were present in samples of pooled house fly eggs (Fig. 1), they were not detected from either the surface or the alimentary canal of any of the house fly F_1_ adults that were sampled. Therefore, no statistics were computed for these two pathogens when included in the model, because all observations had the same response.

We previously reported that *L. monocytogenes* was found in 3 % of wild filth flies [7] and later confirmed that isolated strains belonged to serotype 4b (unpublished data), responsible for most major outbreaks of human listeriosis [39]. However, studies providing evidence of *L. monocytogenes* being vectored by synanthropic filth flies are scarce. The innate immune response elicited by *L. monocytogenes* infections has shown that this bacterium is rapidly detected by the insect, inducing autophagy and inhibiting its intracellular growth to enhance insect survival [40, 41]. Additionally, *L. monocytogenes* are not restricted to localized tissues or specialized cells within the insect [42] and their release from the alimentary canal during metamorphosis may induce both localized and humoral insect immune responses [43], decreasing the overall bacterial population [44]. Thus, the absence of *L. monocytogenes* from house fly F_1_ adults was probably due to the flies’ innate immune response towards this foodborne pathogen. However, the ubiquitous abundance of *L. monocytogenes* in the environment, their ability to survive for long periods of time in acidic soils containing high endogenous microbiota [45], and their capability to attach to environmental surfaces and form biofilms [46] gives them the ability to create new focus of infection that can be used by filth flies to widely spread this pathogen.

*Escherichia coli* O157:H7 was also absent from house fly F_1_ generation adults. While some studies have demonstrated that house flies that ingested high *E. coli* O157:H7 concentrations (10^9^ CFU/ml), retained this pathogen inside the alimentary canal for up to three days [18, 47], some others have reported that immune molecular effectors such as AMPs and lysozymes prevent the proliferation of this pathogen in the fly’s alimentary canal [17]. Thus, the question that *E. coli* O157:H7 is pathogenic to house flies needs to be further investigated. Although *E. coli* O157:H7 was present in samples containing pooled house fly eggs, this pathogen did not persist throughout metamorphosis. This finding was opposite to other studies that have reported the ingestion of non-pathogenic *E. coli* by house fly larva and their persistence throughout pupae and newly emerged adults [48, 49]. However, in this study we did not quantify the amount of *E. coli* O157:H7 present in the larval rearing substrate; hence, the levels of this pathogen that were likely to be ingested by house fly larvae were unknown and probably low enough to avoid their persistence through the house fly life cycle. Nevertheless, the association of synanthropic filth flies with *E. coli* O157:H7 is broadly supported [21, 50–54], strongly suggesting that house flies can indiscriminately disseminate this foodborne pathogen.

*Salmonella* e*nterica* and *C. sakazakii* were the only pathogens present on F_1_ generation adults and only when parental house flies were given food contaminated with medium and high bacterial loads. The analysis of the maximum likelihood estimates of the parameters of this logistic regression model and the model fit statistics are included in Additional file 2(B). As shown by the model fit statistics and AUC values of 0.87 and 0.82 (excellent discrimination) for *S*. e*nterica* and *C. sakazakii*, respectively, our data fit the model in Eq. 2 relatively well. The estimated probability of transmitting these pathogens to any single female adult fly from the F_1_ generation was associated with the bacterial concentration given to parental flies and the body part of the fly.

The presence of *S.* e*nterica* and *C. sakazakii* was 2.4 times more likely on the body surface than in the alimentary canal of newly emerged F_1_ adults (Table 1B). This is in agreement with early studies performed by Radvan [55] who determined that some bacteria including *Bacillus anthracis, B. subtilis, Shigella sonnei,* and non-pathogenic *E. coli* were mainly located on the surface of recently emerged flies. This is probably due to the release of the intestinal content of the larvae into the pupal cavity, one of the changes that take place while the larvae re-organizes into an adult house fly [43, 56].

**Fig. 2:**
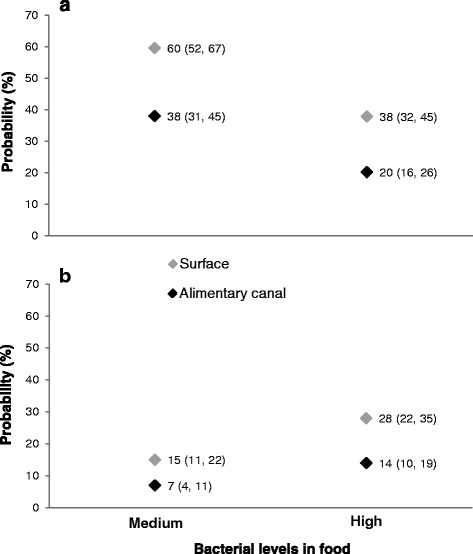
Probability of bacterial transmission to house fly first filial (F_1_) generation adults. **a** *Salmonella enterica* and (**b**) *Cronobacter sakazakii*. Numbers in parenthesis represent lower and upper 95 % confidence limits (CL)

The probability of finding *S. enterica* on a single F_1_ adult house fly was greater than the probability of finding *C. sakazakii* (Fig. 2a, b). When parental flies received food with medium levels of *S. enterica* the probability of finding this pathogen on the surface and in the alimentary canal of a single F_1_ adult fly was 60 and 38 %, respectively. However, this probability decreased 38 and 20 % for the body surface and the alimentary canal, respectively, when parental flies fed from food with high *S. enterica* levels (Fig. 2a). Overall, it was 2.4 times more likely to find *S. enterica* on F_1_ adults after parental flies fed from food containing medium bacterial loads (Table 1B). Even though the presence of *S. enterica* in samples containing pooled house fly eggs was lower than other bacteria evaluated, this pathogen has developed strategies to deal with environmental changes brought on by the whole microbial community of a specific niche [57]. This could allow *Salmonella* to colonize the larval rearing substrate, be re-acquired by the developing larvae and persist through the adult stage.

Contrary to our findings with *S. enterica*, it was 2.2 times more likely to find *C. sakazakii* in a single F_1_ adult house fly after parental flies fed from food contaminated with high levels of this pathogen (Table 1B). The probabilities of finding *C. sakazakii* on the fly’s body surface and in the alimentary canal were 28 and 14 %, respectively, after parental flies fed from highly contaminated food (Fig. 2b). This probability decreased 15 and 7 % for the body surface and the alimentary canal, respectively, when parental flies fed from food contaminated with medium *C. sakazakii* levels. Thus, our results emphasize that pathogen concentration is an important parameter to determine the transmission of bacteria to the house fly progeny. Other authors have also stressed the significance of bacterial concentrations in the transmission of microorganisms since low bacterial inocula are insufficient to colonize the insect and the ingestion of excessive bacteria may be either pathogenic [48, 58, 59] or alter population dynamics or behavior [60].

*Cronobacter sakazakii* and *S. enterica* have probably evolved several mechanisms to evade the fly’s immune system. Bacteria that are associated with food can access the fly’s digestive tract and if they tolerate digestive processes and evade the immune system, they are able to access an environment that allows them to disseminate via regurgitation or defecation [32, 61]. Some ingested pathogenic bacteria can also produce a chronic infection in the host that makes it difficult to distinguish between a pathogenic or beneficial insect-microbe association [32, 62]. If *C. sakazakii* and *S. enterica* provide some benefit to synanthropic filth flies needs to be studied further. Examples of beneficial facultative symbionts by several arthropods include *Serratia symbiotica* in the pea aphid, *Acyrthosiphon pisum* (Hemiptera: Aphididae), which confers resistance against natural enemies such as parasitic wasps [63–65], and *Hamiltonella defensa* in whiteflies *Bemisia tabaci* (Hemiptera: Aleyrodidae) that increases the development and fitness of the host [66]. Consequently, the transmission mechanisms of both *C. sakazakii* and *S. enterica* need to be studied through more than one generation of flies to elucidate the type of associations these bacteria can potentially establish with these insects. Additionally, the interactions of these foodborne pathogens with other microorganisms present in flies need to be further explored. Understanding the type of associations that synanthropic flies establish with foodborne pathogens will help to elucidate transmission mechanisms as well as possible ways to mitigate the spread of foodborne pathogens.

It is important to mention that there is zero tolerance for the presence of *S. enterica*, *C. sakazakii*, *E. coli* O157:H7*,* or *L. monocytogenes* in foods. The mere presence of any of these four foodborne pathogens deems the food to be adulterated. Because the concentration of these pathogens in foods is usually not quantified, it is difficult to associate the three levels of contaminated food given to flies, to contamination concentrations of these pathogens in foods. Interestingly, this study demonstrated that adult house flies feeding from food contaminated with levels of bacteria as low as 100 cells/ml are able to transfer ingested pathogens to their progeny. Even though food can become contaminated at any point during production, the presence of pests, such as flies, increases the potential risk of pathogen transmission. Synanthropic filth flies that feed from any level of contaminated food are able to disseminate pathogens indiscriminately, not only mechanically or through regurgitation and defecation but also to their progeny, greatly increasing their vector potential.

To better protect public health, it is important to highlight the need for effective preventative measures that minimize the hazard posed by pests that may come in contact with food or food-contact surfaces and utensils. The implementation of pest control programs is one of the frequently and highly recommended measures to avoid the indirect transmission of foodborne pathogens by synanthropic insects like flies. The effectiveness of the program should be constantly monitored and filthy breading sites should be eliminated. By targeting control measures towards synanthropic filth flies, the potential transmission of foodborne pathogens can be interrupted, contributing to the prevention of future foodborne illness outbreaks.

## Conclusion

In this study, we demonstrated that adult house flies that fed from food contaminated with low, medium, and high levels of *S. enterica*, *C. sakazakii*, *E. coli* O157:H7 or *L. monocytogenes* transmit these pathogens to their eggs. *Salmonella enterica* and *C. sakazakii* were further transmitted to F_1_ generation house fly adults, and they were more commonly found on the surface than in the alimentary canal of newly emerged house flies. Results from this research emphasize the public health significance and the regulatory importance of the presence of flies in food and food facilities.

## Methods

### House fly source

House fly (*M. domestica*) puparia were obtained from Spider Pharm, Inc. (Yarnell, AZ) and placed in plastic cages inside a Percival growth chamber at 30 °C and 16:8 h light:dark (L:D) photoperiod until eclosion. Emerged house flies were fed with a dry mixture of 1:1 granulated sugar and powdered milk. Cotton balls soaked in autoclaved water were also provided as a water source. Adult house flies (2-4 days old) were immobilized by placing the plastic cages at -30 °C for 5-7 min. Groups of approximately 40 adults (mixed sex) were transferred to autoclaved wide-mouth quart Mason glass jars. A disinfected 6-inch square piece of fiberglass window screen (New York Wire, Hanover, PA) was placed on top of each jar and secured with a rubber band. All glass jars were kept in the Percival growth chamber under the same conditions described above.

### Preparation of contaminated food

Four bacterial foodborne pathogens (*S. enterica*, *C. sakazakii*, *E. coli* O157:H7*,* and *L. monocytogenes*) were used in our study. Information about bacterial strains, serotypes, and their origin is specified in Additional file 4. Bacterial strains were reconstituted from 30 % glycerol stock cultures, plated on Trypticase Soy Agar (TSA; Oxoid, Cambridge, UK), and incubated at 37 °C overnight. Stock suspensions of each bacterium were prepared by scraping bacterial cells from overnight cultures and adding them to buffered peptone water (BPW; Difco, Becton, Dickinson and Company, Sparks, MD). The optical density of the stock suspension was measured at 600 nm (OD_600_) using a GENESYS™ 20 Spectrophotometer (Thermo Fisher Scientific, Rochester, NY), and the bacterial concentration was calculated assuming that 0.1 OD_600_ = 10^8^ bacterial cells/ml [67, 68]. A known volume of the stock bacterial suspension was added to a known volume of liquid fly food (18 g of dried powdered milk, 4 g of sugar, 2 g of protein powder, and 200 ml sterile distilled water) to obtain final bacterial concentrations of 10^8^, 10^4^, and 10^2^ CFU/ml of each foodborne pathogen.

### Adult house fly feeding bioassay

For each pathogen, approximately ten ml of fly food with the corresponding level of bacteria was added to three autoclaved cotton balls that were previously placed in the base of a sterile 60 mm diameter Petri dish. Fly food with no bacteria was used to feed the control groups. Fly food was given to parental house flies by inverting the Petri dish onto the mesh screen on top of each glass jar (see Additional file 5(A)), replacing with the corresponding fresh food after 18-20 h. Jars were kept in the Percival growth chamber under the same conditions described before and adult house flies were allowed to mate and feed *ad libitum* for a total of 30-32 h. Although the level of bacterial contamination of the fly food provided to parental house flies was known, the amount of bacteria actually ingested by adult house flies was not quantified. Thus, fly food containing final bacterial concentrations of 10^8^, 10^4^, and 10^2^ CFU/ml will be referred hereinafter as high, medium, and low, respectively. After completing the feeding time, the Petri dish and cotton balls were removed and the mesh screen was thoroughly cleaned and disinfected with 70 % ethanol before adding the oviposition substrate.

### Collection of house fly eggs

To create an oviposition substrate, several pieces of dehydrated beef liver (approximately 1 cubic inch and hydrated overnight) were placed on top of the mesh screen of each glass jar and covered with the lid of a sterile Petri dish to prevent dehydration (see Additional file 5(B)). Once fly eggs were visible on the surface of the liver, the glass jars were removed from the Percival growth chamber and clusters of approximately 100 eggs (laid by several females) were carefully removed using autoclaved forceps. To remove microbiota from the outer surface of the eggs, each cluster of eggs was transferred to a two ml tube with 70 % ethanol for 1 min, then submersed in 0.05 % bleach for 1 min, and finally rinsed three times with autoclaved distilled water (see Additional file 5(C)). One-hundred μl aliquots of water from the last rinse were plated on chromogenic media specific for the target foodborne pathogen (see Additional file 4). Surface-disinfected house fly eggs were divided in two groups approximately equal in number (~40-50). To assess the presence of pathogens, the first group of pooled eggs was added to one ml of enrichment media specific for each bacterial pathogen and incubated accordingly (see Additional file 4). The second group of eggs was added to a larval rearing substrate and allowed to hatch and complete their life cycle to evaluate the presence of foodborne pathogens in adult house flies of the F_1_ generation.

### Validation that parental house fly adults ingested bacteria

After eggs were collected, glass jars containing parental house flies were placed at -20 °C for 5-7 min until flies were immobilized. Immobilized flies were then transferred to a disposable Petri dish containing 70 % alcohol for 2 min. Using a dissecting scope, three adult female house flies were randomly sub-sampled per each glass jar (n = 48 per each foodborne bacterium) and individually transferred to an autoclaved two ml tube to be surface-disinfected and their alimentary canals dissected as described by Pava-Ripoll*, et al.* [27]. The alimentary canals of maternal house flies were individually evaluated for the presence of the target bacteria as described in sections below.

### House fly F_1_ offspring rearing procedure

The larval rearing substrate was prepared by pre-mixing dry ingredients (1 cup of autoclaved alfalfa pellets, 1 cup of autoclaved wheat bran, 1 cup of autoclaved bone meal, 1 cup of autoclaved poultry litter, 1/3 cup of dried milk powder, and 1 teaspoon of Brewer’s yeast) and adding 4 ½ cups of autoclaved tap water. Half cup of the prepared larval rearing substrate was added to individual plastic containers and then the group of surface-disinfected house fly eggs was added to the substrate using a disposable plastic pipette. The container was then nested in a larger plastic container that was approximately 1/8^th^ filled with autoclaved sand to give fly larvae a dry place to pupate (see Additional file 5(D)). The rearing chambers were covered with an autoclaved paper towel, secured with a rubber band and placed in a Percival growth chamber at 32-35 °C and 16:8 h L:D photoperiod until pupation (approximately 4-5 days; see Additional file 5(E)). Using a disposable 1000 μl sterile pipette tip the larval substrate was gently mixed every day to inhibit mold growth. House fly pupae from each rearing chamber were carefully separated from the sand using sterile forceps and transferred to an extra-deep sterile, disposable Petri dish (Fisherbrand, Thermo Fisher Scientific, Rochester, NY) to allow F_1_ adults to emerge avoiding cross-contamination with the larval rearing substrate (see Additional file 5(F)). Petri dishes containing pupae were kept in the Percival growth chamber under same conditions until emergence of F_1_ generation adults (approximately 2-3 additional days; see Additional file 5(G)).

### Collection of female F_1_ generation house fly adults

Recently emerged (0-1 days old) F_1_ adults were immobilized by placing extra-deep Petri dishes at -20 °C for 5-7 min. Under a dissecting scope, three females were randomly sub-sampled per each Petri dish (n = 48 per each foodborne bacterium) and individually transferred to autoclaved two ml tubes containing one ml of enrichment media specific for the target pathogen (see Additional file 4) to collect microbiota from the surface of the newly emerged house fly. Each house fly was then removed from the enrichment media, surface-disinfected and their alimentary canals aseptically dissected as described by Pava-Ripoll*, et al.* [27]. Tubes with enrichment media containing microbiota from the surface (s) and the alimentary canal (ac) of each F_1_ adult house fly were incubated at times and temperatures recommended for each bacterial pathogen (see Additional file 4).

### Detection and isolation of the target bacteria

Enriched samples were assessed for the presence/absence of the target bacteria using a combined molecular and culture approach. The molecular approach was performed using a commercial PCR cycler/detector system (BAX® System Q7, DuPont Qualicon, Wilmington, DE) and assay kits specific for each bacteria (see Additional file 4) following manufacturer’s instructions and as described by Pava-Ripoll*, et al.* [27]. Each assay kit contains PCR-ready tablets with an intercalating dye that emits a fluorescence signal when binding to the target double-stranded DNA. The signal is detected by the PCR system and interpreted by the software as positive or negative. The culture approach was performed by plating ten μl of the enrichment media of PCR-positive samples on chromogenic media specific for each bacterium (see Additional file 4) until pure colonies were obtained. The culture approach was performed to confirm that isolated pathogens were the same strains given to parental house flies. Isolated *S. enterica*, *L. monocytogenes*, and *E. coli* O157:H7 were confirmed through pulsed-field gel electrophoresis (PFGE), following the protocols described by PulseNet and only using primary enzyme restriction [69, 70]. Isolated *C. sakazakii* was confirmed by polymerase chain reaction (PCR) amplification of the diguanylate cyclase (*cgcA*) gene using primers Cmstu-825 F and Csak-1317R as described by Carter*, et al.* [71]. Amplicons of expected size (463 bp) of the singleton PCR reaction were purified and sequenced by Retrogen, Inc. (San Diego, CA) and sequence files were imported into Sequencher 5.0 (GeneCodes, Ann Arbor, MI) to be processed and assembled. Contigs were exported and aligned using the CLUSTALX software (Lasergene, Madison, WI) and aligned sequences were used to generate a variance table report (Sequencher 5.0) where nucleotide bases of each sequence were compared to the reference *C. sakazakii* sequence. Four to five randomly selected PCR-negative samples were also plated on specific chromogenic media to confirm the absence of the target pathogen.

### Experimental design

This experiment was set up as a completely randomized design and was performed at four different times with a one-month interlude. One foodborne pathogen (*S. enterica*, *C. sakazakii*, *E. coli* O157:H7*,* or *L. monocytogenes*) and fly food with three levels of bacterial contamination (high, medium, and low) plus a control, consisting of fly food with no bacteria, were evaluated each time. Each treatment combination (foodborne pathogen by levels of contaminated food) was replicated four times. Thus, 16 glass jars containing parental generation of adult flies were prepared each time the experiment was run. The presence/absence of the target bacterium was assessed as follows: a) from the alimentary canals of three parental females that were randomly sub-sampled per replicate (n = 48 per each foodborne pathogen); b) from pooled house fly eggs laid by several parental females (n = 16 per each foodborne pathogen); and c) from body surfaces and alimentary canals of three F_1_ female house flies that were randomly sub-sampled per replicate (n _surface_ = 48 and n _alimentary canal_ = 48 per each foodborne pathogen).

### Statistical analysis

We used the SAS logistic regression procedure (PROC LOGIT; SAS Institute Inc., 2005) to predict the probability of bacterial contamination to house fly eggs and to the surface and the alimentary canal of F_1_ female adults. The presence/absence of foodborne pathogens was the categorical dichotomous response variable and its relationship with the predictor variables was analyzed using the two full logistic probability models described in Eq. 1 (for house fly eggs) and Eq. 2 (for F_1_ generation house fly adults).

1 $$ \begin{array}{l}\mathrm{Logit}\ {\left(\mathrm{P}\right)}_{\mathrm{eggs}}={\upbeta}_0+{\upbeta}_1* foodborne\  pathogen+{\upbeta}_2* bacterial\  levels\  of\  contaminated\  food+{\upbeta}_3* foodborne\  pathogen\ *\\ {} bacterial\  levels\  of\  contaminated\  food\end{array} $$

2 $$ \mathrm{Logit}\ {\left(\mathrm{P}\right)}_{\mathrm{F}1}={\upbeta}_0\kern0.5em +\kern0.5em {\upbeta}_1* bacterial\  levels\  of\  contaminated\  food+{\upbeta}_2* house\  fly's\  body\  part $$

Where logit (P) = ln [P/1-P], ln is the natural log, *P* is the probability of the presence of bacteria, β_0_ is the *P* intercept, β_i_ are regression coefficients. The predictor variables for the probabilistic model of house fly eggs (Eq. 1) were the type foodborne pathogen, the level of bacterial contamination of the food given to parental house flies and their interaction. The predictor variables for the probabilistic model of F_1_ generation of house fly adults were the level of bacterial contamination of the food given to parental house flies and the fly’s body part (surface and alimentary canal) and the model was analyzed by each foodborne pathogen. The stepwise selection method with analysis of maximum likelihood estimates based on a Wald Chi-square *p* value <0.05 was used to determine the best probability model. The receiver operating characteristics (ROC) curve was used as a measurement of the goodness-of-fit of the model. The ROC curve quantifies the power of the predicted values using the area under the ROC curve (AUC). AUC values >0.7 are considered acceptable discrimination, >0.8 are considered excellent discrimination and >0.9 are considered outstanding discrimination [72].

## Additional files

Additional file 1:
**Pulsed-field gel electrophoresis (PFGE) profiles.** The PFGE fingerprinting shows an indistinguishable pattern between the bacterial strains used to feed parental flies and the bacterial colonies isolated from the alimentary canal of parental flies (Pac), house fly eggs (e), and the surface (s) and alimentary canal (ac) of adult flies from the first filial (F_1_) generation. Profiles were obtained from (A) *Salmonella enterica* serotype Schwarzengrund (strain SAL3542; PFGE PulseNet pattern JM6X01.0289); (B) enterohemorrhagic *Escherichia coli* O157:H7 (strain ESC0786; PFGE PulseNet pattern EXHX01.0125); and (C) *Listeria monocytogenes* serotype 4b (strain LIS0150; PFGE PulseNet combined pattern GX6A16.0059_GX6A12.1652). Additional file 2:
**Analysis of Maximum Likelihood Estimates (MLE) of the logistic regression model.** (A) house fly eggs and (B) house fly first filial (F_1_) generation of adults. Additional file 3:
**Scanning Electron Microscopy (SEM) of surface-disinfected house fly (** ***Musca domestica*** **) eggs (A) house fly egg; (B) the hatching line, with distinct curved rib-like thickenings; and (C) adhesive fluid on the egg surface.**
Additional file 4:
**Information about foodborne bacteria, culture media, incubation conditions, and PCR-based kits used in this study. **
Additional file 5:
**Experimental setup**. (A) feeding of the parental population of house flies, (B) oviposition substrate, (C) collected house fly eggs, (D) surface-disinfected eggs placed in the larval rearing substrate, (E) house fly larval rearing container, (F) transfer of house fly pupae to plates, (G) emergence of first filial (F_1_) generation of house fly adults.
