# Supplementary material for: Ingested Salmonella enterica, Cronobacter sakazakii, Escherichia coli O157:H7, and Listeria monocytogenes: transmission dynamics from adult house flies to their eggs and first filial (F1) generation adults
Source: BMC Microbiol. 2015 Jul 31;15:150. doi: 10.1186/s12866-015-0478-5 (PMC4520200; doi:10.1186/s12866-015-0478-5)
Supplement: Additional file 1: — Pulsed-field gel electrophoresis (PFGE) profiles. The PFGE fingerprinting shows an indistinguishable pattern between the bacterial strains used to feed parental flies and the bacterial colonies isolated from the alimentary canal of parental flies (Pac), house fly eggs (e), and the surface (s) and alimentary canal (ac) of adult flies from the first filial (F1) generation. Profiles were obtained from (A) Salmonella enterica serotype Schwarzengrund (strain SAL3542; PFGE PulseNet pattern JM6X01.0289); (B) enterohemorrhagic Escherichia coli O157:H7 (strain ESC0786; PFGE PulseNet pattern EXHX01.0125); and (C) Listeria monocytogenes serotype 4b (strain LIS0150; PFGE PulseNet combined pattern GX6A16.0059_GX6A12.1652). [file 12866_2015_478_MOESM1_ESM.pdf]

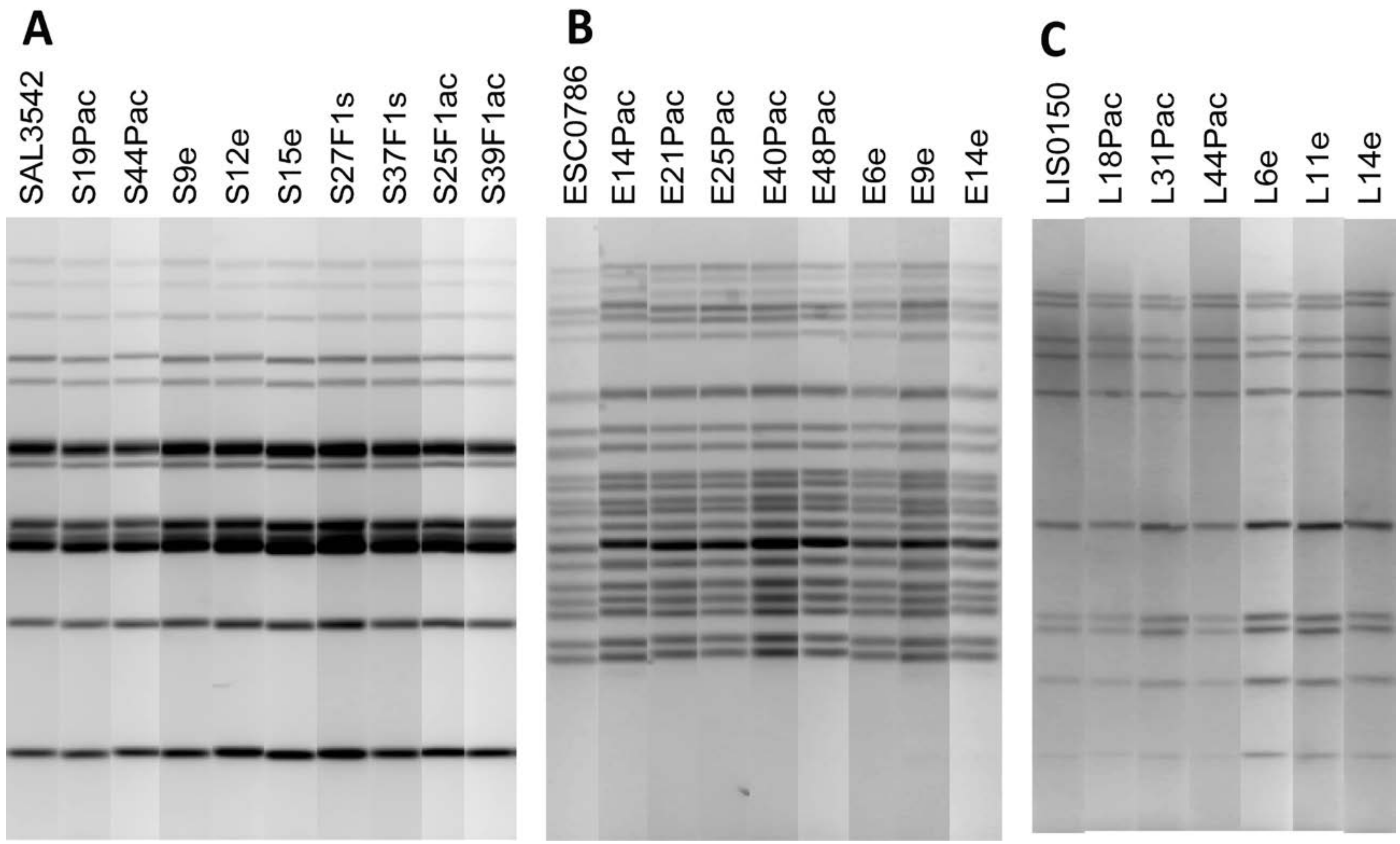

Additional file 1 - Pulsed-field gel electrophoresis (PFGE) profiles

The PFGE fingerprinting shows an indistinguishable pattern between the bacterial strains used to feed parental flies and the bacterial colonies isolated from the alimentary canal of parental flies (Pac), house fly eggs (e), and the surface (s) and alimentary canal (ac) of adult flies from the first filial (F_1) generation. Profiles were obtained from (A) *Salmonella enterica* serotype Schwarzengrund (strain SAL3542; PFGE PulseNet pattern JM6X01.0289); (B) enterohemorrhagic *Escherichia coli* O157:H7 (strain ESC0786; PFGE PulseNet pattern EXHX01.0125); and (C) *Listeria monocytogenes* serotype 4b (strain LIS0150; PFGE PulseNet combined pattern GX6A16.0059_GX6A12.1652).
