# Supplementary material for: Ingested Salmonella enterica, Cronobacter sakazakii, Escherichia coli O157:H7, and Listeria monocytogenes: transmission dynamics from adult house flies to their eggs and first filial (F1) generation adults
Source: BMC Microbiol. 2015 Jul 31;15:150. doi: 10.1186/s12866-015-0478-5 (PMC4520200; doi:10.1186/s12866-015-0478-5)
Supplement: Additional file 2: — Analysis of Maximum Likelihood Estimates (MLE) of the logistic regression model. (A) house fly eggs and (B) house fly first filial (F1) generation of adults. [file 12866_2015_478_MOESM2_ESM.pdf]

Additional file 2 - Analysis of Maximum Likelihood Estimates (MLE) of the logistic regression model

(A) house fly eggs and (B) house fly first filial (F_1) generation of adults .

	Parameter	df	Estimate (β)	SE	Wald X^2	*p* value
**A) House fly eggs***						
	Intercept		-3.5312	340.4	0.0001	0.9917
	**Bacterial levels in food**					
	Low	1	4.0345	340.4	0.0001	0.9905
	Medium	1	5.8210	340.4	0.0003	0.9864
	High	1	5.1965	340.4	0.0002	0.9878
	Control	1	-15.0520	1021.2	0.0002	0.9882
	**Foodborne pathogen**					
	C. sakazakii	1	1.3926	0.5951	5.4761	0.00193
	E. coli O157:H7	1	-0.3518	0.4093	0.7389	0.3900
	L. monocytogenes	1	0.3052	0.4506	0.4589	0.4981
	S. enterica	1	-1.3460	0.3954	11.5897	0.0007
**B) House fly F_1 adults**						
S. enterica*						
	Intercept	1	-9.5199	38.1	0.0006	0.9803
	**Bacterial levels in food**					
	Low	1	-9.0274	693	0.0002	0.9896
	Medium	1	9.4699	386.1	0.0006	0.9804
	High	1	8.5848	386.1	0.0005	0.9823
	Control	1	-9.0274	1005.1	0.0001	0.9928
	**Body part**					
	Alimentary canal	1	-0.4397	0.0889	24.4646	<0.0001
	Surface	1	0.4397	0.0889	24.4646	<0.0001
C. sakazakii**						
	Intercept	1	-10.4840	535.1	0.0004	0.9844
	**Bacterial levels in food**					
	Low	1	-8.7161	960.6	0.0001	0.9928
	Medium	1	8.3305	535.1	0.0002	0.9876
	High	1	9.1017	535.1	0.0003	0.9864
	Control	1	-8.7161	1437.0	0.0001	0.9928
	**Body part**					
	Alimentary canal	1	-0.4393	0.1162	14.2930	0.0002
	Surface	1	0.4393	0.1162	14.2930	0.0002

SE= Standard error.

***Summary of stepwise selection (n=64):** Bacterial concentration: df=3, X^2= 76.294, p<0.0001; Foodborne pathogen: df=3, X^2= 15.4601, p=0.0015; Bacterial concentration* Foodborne pathogen: df=9, Wald X^2=0, p=1. **Model fit statistics:** Area Under the ROC Curve

(AUC)=0.8889, AIC: 220.772, likelihood ratio test= 110.8146, df=6, P<0.0001, Score= 84.9562, Wald= 18.1559, R^2=0.8230, Max-rescaled R^2=0.8509, Somers's D_{xy} = 0.8889.

****Summary of stepwise selection (n=96):** Bacterial concentration: df=3, X^2=139.8853, p<0.0001; body part: df=1, X^2=24.9831, p<0.0001. **Model fit statistics:** Area Under the ROC Curve (AUC)=0.8732, AIC=961.115, likelihood ratio test=215.9087, df=4, p<0.0001, Score=160.5856, Wald=45.3982, R^2=0.8945, Max-rescaled R^2=0.8945, Somers's D_{xy}=0.746.

*****Summary of stepwise selection (n=96):** Bacterial concentration: df=3, X^2=54.6138, p<0.0001; body part: df=1, X^2=14.8349, p=0.0001. **Model fit statistics:** Area Under the ROC Curve (AUC)=0.8239, AIC=612.986, likelihood ratio test=92.7704, df=4, p<0.0001, Score=68.4558, Wald=23.5347, R^2=0.6195, Max-rescaled R^2=0.6206, Somers's D_{xy}=0.648.
