# Supplementary material for: Ingested Salmonella enterica, Cronobacter sakazakii, Escherichia coli O157:H7, and Listeria monocytogenes: transmission dynamics from adult house flies to their eggs and first filial (F1) generation adults
Source: BMC Microbiol. 2015 Jul 31;15:150. doi: 10.1186/s12866-015-0478-5 (PMC4520200; doi:10.1186/s12866-015-0478-5)
Supplement: Additional file 3: — Scanning Electron Microscopy (SEM) of surface-disinfected house fly ( Musca domestica ) eggs (A) house fly egg; (B) the hatching line, with distinct curved rib-like thickenings; and (C) adhesive fluid on the egg surface. [file 12866_2015_478_MOESM3_ESM.pdf]

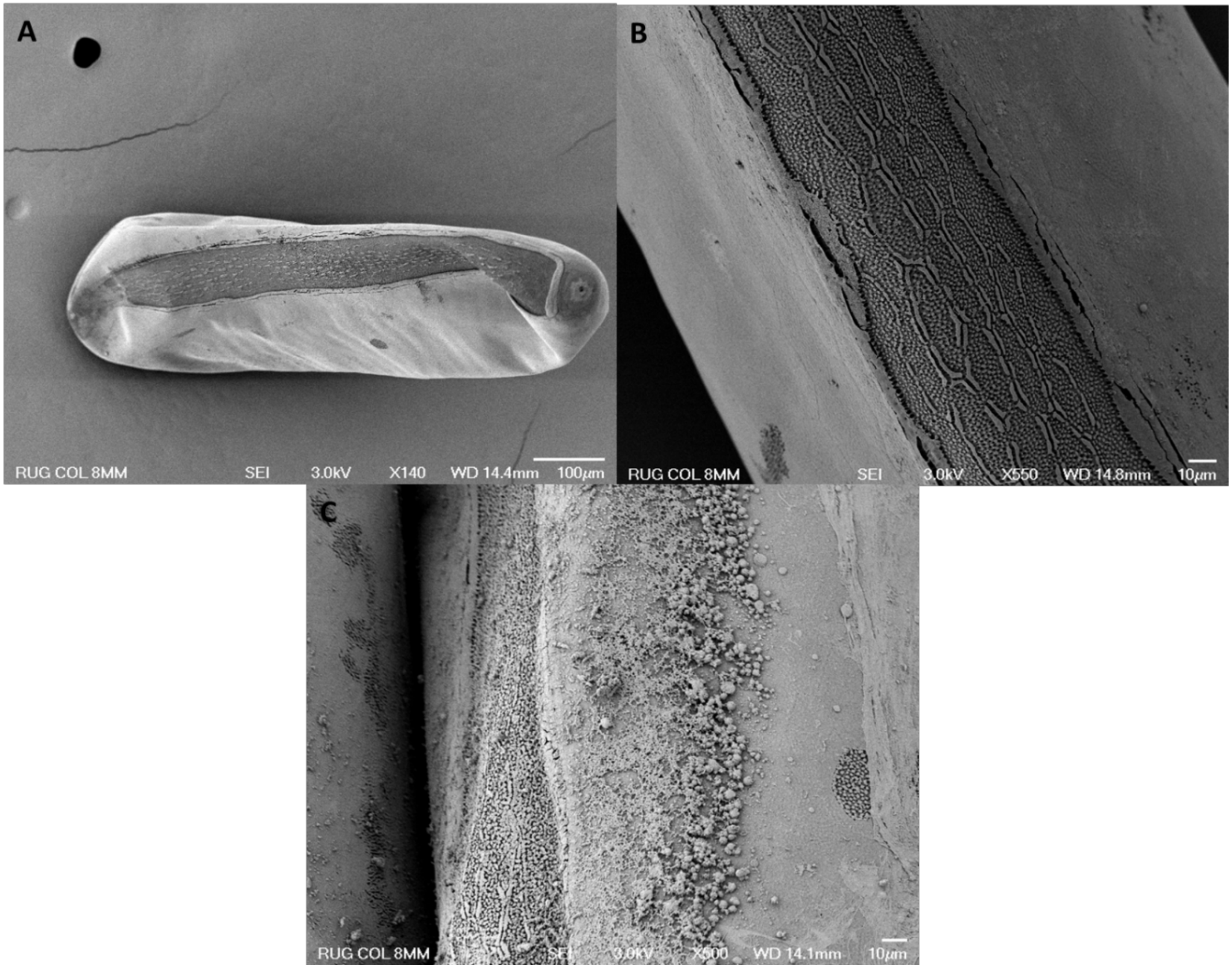

Additional file 3 - Scanning Electron Microscopy (SEM) of surface-disinfected house fly (*Musca domestica*) eggs

(A) house fly egg; (B) the hatching line, with distinct curved rib-like thickenings; and (C) adhesive fluid on the egg surface

Protocol: Surface-disinfected house fly eggs were pre-fixed in 1% glutaraldehyde in PBS, pH 7.2 for 18 h at 4°C. The eggs were then gently washed with three changes of PBS, pH 7.2 at 4°C (15 min. wash times) and post-fixed in 1% osmium tetroxide in PBS, for 24 h at 4°C. The eggs were once again washed with three changes of PBS at 4°C and then dehydrated with a graded series of ethanol (10 to 100%, 15 min at 4°C per step, with 3 exchanges of 100% ethanol), and critical point-dried (purge time, 15 min in liquid $C0_2$, using an Autosamdri 815B (Tousimis Research Corp., Rockville, MD).The eggs were mounted onto aluminum stubs; and sputter coated with gold in argon, for 30 secs. at 50 mA, using a Denton Vacuum Desk V series sputter coater (Moorestown, NJ). Specimens were viewed in a JSM-6701F field emission SEM operated at an accelerating voltage of 3 kV.
