# Supplementary material for: Ingested Salmonella enterica, Cronobacter sakazakii, Escherichia coli O157:H7, and Listeria monocytogenes: transmission dynamics from adult house flies to their eggs and first filial (F1) generation adults
Source: BMC Microbiol. 2015 Jul 31;15:150. doi: 10.1186/s12866-015-0478-5 (PMC4520200; doi:10.1186/s12866-015-0478-5)
Supplement: Additional file 4: — Information about foodborne bacteria, culture media, incubation conditions, and PCR-based kits used in this study. [file 12866_2015_478_MOESM4_ESM.pdf]

Additional file 4 - Information about foodborne bacteria, culture media, incubation conditions, and PCR-based kits used in this study

Foodborne pathogen*			Enrichment Media		PCR-based (BAX) Kit	Chromogenic Media (37°C for 24-48 h)
**Genus and species**	**Serovar/Strain**	**Origin**	**Primary enrichment / Incubation conditions**	**Secondary enrichment / Incubation conditions**		
Salmonella enterica	Schwarzengrund / SAL3542	Alimentary canal of *Lucilia cuprina*	BPW (pre-warmed at 42°C) / Recirculating water bath at 42.5°C for 22-24 h	Transfer 100 µl of primary enriched sample to 400 µl of Brain-heart infusion (BHI) broth (pre-warmed at 37°C) / 37°C for 3 h	*Salmonella* 2 (standard assay)	*Salmonella* chromogenic agar with selective supplement (Oxoid, Cambridge, UK)
Listeria monocytogenes	4b / LIS0150	Alimentary canal of *Lucilia cuprina*	24 *Listeria* Enrichment Broth (24 LEB) with selective supplement (freshly prepared and at room-temperature; Oxoid, Cambridge, UK) / 37°C for 44±5 h	Not applicable	*L. monocytogenes* (24E assay)	RAPID' *L.mono* agar (Bio-Rad Laboratories, Hercules, CA)
Cronobacter sakazakii	O2 / Md5g *aka* ENTB0353	Alimentary canal of *Musca domestica*	BPW with 10 mg/L of Novobiocin (pre-warmed at 37°C) / 37°C for 22-26 h	Transfer 100 µl of primary enriched sample to 400 µl of BHI broth (pre-warmed at 37°C) / 37°C for 3 h	*E. sakazakii* (standard assay; used to detect *C. sakazakii*)	Brilliance *Cronobacter* agar (*aka* Druggan-Forsythe-Iversen formulation-DFI; Oxoid, Cambridge, UK)
Escherichia coli	O157:H7 / ESC0786	Spinach	BAX System *E. coli* O157:H7 MP media broth (pre-warmed at 42°C; DuPont Qualicon, Wilmington, DE) / 42±1°C for 18-24 h	Not applicable	*E. coli* O157:H7 MP (standard assay)	R & F *E. coli* O157:H7 chromogenic plating medium (R & F Laboratories, Downers Grove, IL)

*Bacterial strains were provided by U.S. Food and Drug Administration, Center for Food Safety and Applied Nutrition (FDA/CFSAN)
