# Supplementary material for: Ingested Salmonella enterica, Cronobacter sakazakii, Escherichia coli O157:H7, and Listeria monocytogenes: transmission dynamics from adult house flies to their eggs and first filial (F1) generation adults
Source: BMC Microbiol. 2015 Jul 31;15:150. doi: 10.1186/s12866-015-0478-5 (PMC4520200; doi:10.1186/s12866-015-0478-5)
Supplement: Additional file 5: — Experimental setup. (A) feeding of the parental population of house flies, (B) oviposition substrate, (C) collected house fly eggs, (D) surface-disinfected eggs placed in the larval rearing substrate, (E) house fly larval rearing container, (F) transfer of house fly pupae to plates, (G) emergence of first filial (F1) generation of house fly adults. [file 12866_2015_478_MOESM5_ESM.pdf]

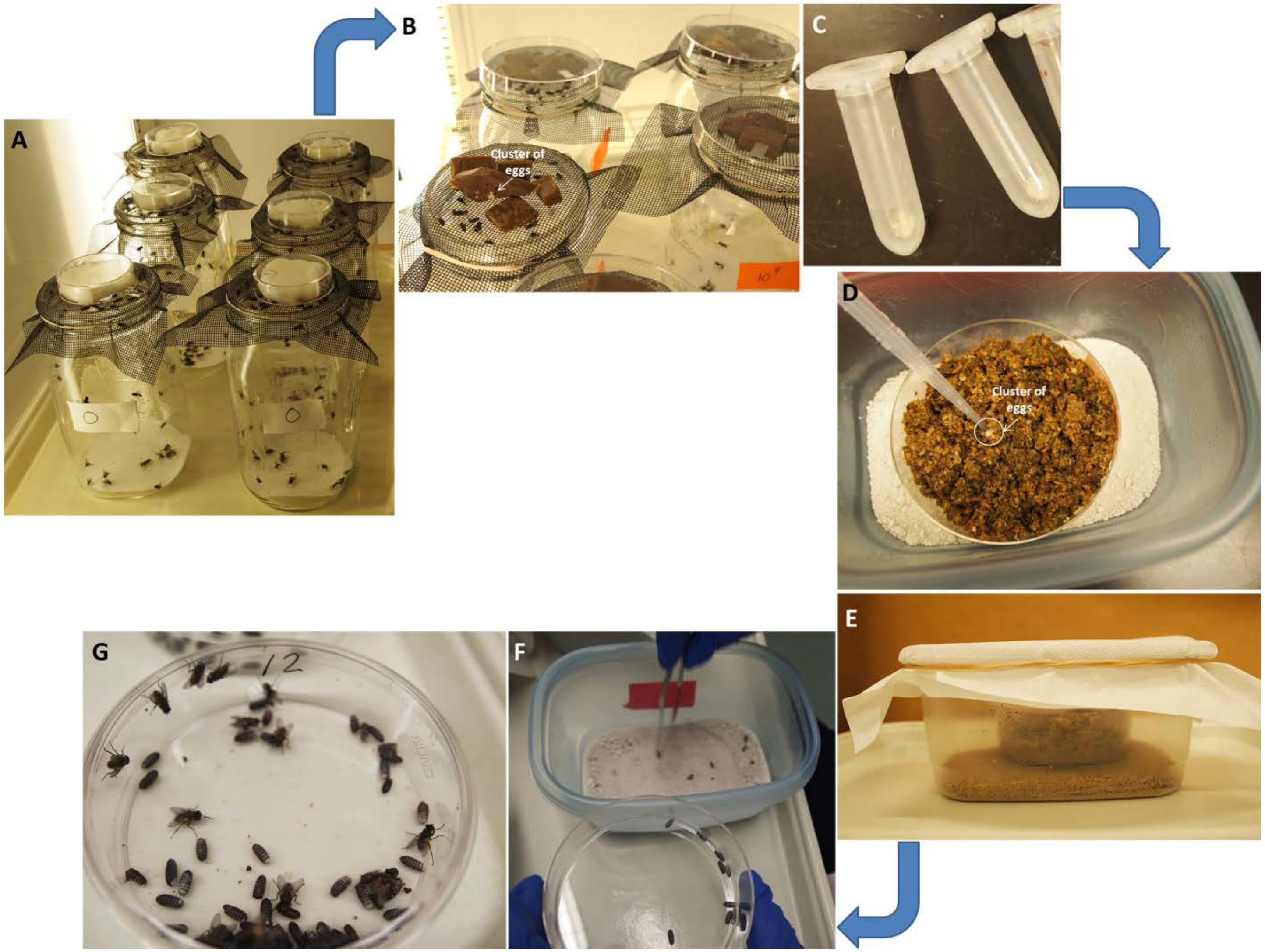

Additional file 5 – Experimental setup

(A) feeding of the parental population of house flies, (B) oviposition substrate, (C) collected house fly eggs, (D) surface-disinfected eggs placed in the larval rearing substrate, (E) house fly larval rearing container, (F) transfer of house fly pupae to plates, (G) emergence of first filial (F_1) generation of house fly adults.
